# State and socio-demographic group variation in out-of-pocket expenditure, borrowings and Janani Suraksha Yojana (JSY) programme use for birth deliveries in India

**DOI:** 10.1186/1471-2458-12-1048

**Published:** 2012-12-05

**Authors:** Hanimi Reddy Modugu, Manish Kumar, Ashok Kumar, Christopher Millett

**Affiliations:** 1 South Asia Network for Chronic Disease (SANCD), Public Health Foundation of India (PHFI), C1/52, First Floor, Safdarjung Development Area, New Delhi 110 016, India; 2 The Vistaar Project, IntraHealth International Inc., New Delhi, India; 3 The Vistaar Project, IntraHealth International, Inc., Ranchi, Jharkhand, India; 4 School of Public Health, Imperial College, London, United Kingdom

## Abstract

**Background:**

High out-of-pocket-expenditure (OOPE) deters families from seeking skilled/institutional care. *‘Janani Suraksha Yojana (JSY),* a conditional cash transfer programme launched in 2005 to mitigate OOPE and to promote institutional deliveries among the poor, is part of Government of India’s efforts to achieve Millennium Development Goals (MDGs) 4 and 5. The objective of this study is to estimate variations in OOPE for normal/caesarean-section deliveries, JSY-programme use and delivery associated borrowings - by states and union territories, and socio-demographic profiling of families, in India.

**Methods:**

Secondary analysis of data from the District Level Household Survey (DLHS-3), 2007–08. Mean and median OOPE, percentage use of JSY and percentage of families needing to borrow money to pay for delivery associated expenditure was estimated for institutional and home deliveries.

**Results:**

Half (52%) of all deliveries in India occurred at home in 2007/08. OOPE for women having institutional deliveries remained high, with considerable variation between states and union territories. Mean OOPE (SD) of a normal delivery in public and private institution respectively in India were Rs. 1,624 and Rs. 4,458 and for a caesarean-section it was Rs. 5,935 and Rs. 14,276 respectively. There was considerable state-level variation in use of the JSY programme for normal deliveries (15% nationally; ranging from 0% in Goa to 43% in Madhya Pradesh) and the percentage of families having to borrow money to pay for a caesarean-section in a private institution (47% nationally; ranging from 7% in Goa to 69% in Bihar). Increased literacy and wealth were associated with a higher likelihood of an institutional delivery, higher OOPE but no major variations in use of the JSY.

**Conclusions:**

Our study highlights the ongoing high OOPE and impoverishing impact of institutional care for deliveries in India. Supporting families in financial planning for maternity care, additional investment in the JSY programme and strengthening state level planning are required to increase the proportion of institutional deliveries.

## Background

In India, high out-of-pocket expenditure (OOPE) is one of the main deterrents to seeking skilled/institutional care [1,2]. With OOPE on health increasing as a proportion of household expenditure [3], poor families (particularly the two lowest quintiles) are becoming particularly vulnerable when these expenditures exceed their capacity to pay [4]. In 2004–05, about 39 million Indians (around 4% of population) fell into poverty due to OOPEs on health care [5,6], including maternal health care.

Since maternal mortality is generally lower where a higher proportion of deliveries are conducted by skilled birth attendants, experts feel this should be a central element of any policy or programme that aims to reduce maternal deaths [7]. The maternal mortality ratio (MMR) in India declined substantially from 398 per 100,000 live births in 1997–98 [8] to 212 per 100,000 live births in 2007-09 [9], with under-five mortality rate (U5MR) declining from 109 per 1,000 live births in 1992–93 [10] to 74 per 1,000 live births in 2005–06 [11]. The proportion of institutional deliveries increased from 39% in 2005–06 [11] to 73% in 2009 [12]. However, India is still far from achieving its Millennium Development Goals (MDGs) 4 and 5 (38 deaths per 1,000 live births for child mortality and less than 100 deaths per 100,000 births for maternal mortality) and universal institutional delivery care, by 2015 [13].

In 2005, Government of India launched *‘Janani Suraksha Yojana (JSY)’* programme', a safe motherhood intervention under the National Rural Health Mission (NRHM), with the objective of reducing maternal and neo-natal mortality by promoting institutional deliveries among the poor [13,14]. JSY is the largest conditional cash transfer programme in the world in terms of number of beneficiaries and constitutes a major Indian health care programme [13,15]. It is a centrally sponsored demand generation programme for 100 percent cash transfer to incentivise women/family to give birth in health facilities. Even though JSY is a centrally sponsored scheme, its implementation differs across the states and union territories [14]. Within five years JSY has made substantial strides, with the number of beneficiaries increasing from 0.74 million in 2005–06 to 10 million in 2009–10 [16], thus covering around 40 percent of total deliveries in the country. Its budgetary allocation has also increased from US$ 8.5 million in 2005–06 to US$ 275 million in 2008–09 [13].

Janani Suraksha Yojana (JSY) programme guidelines

*According to JSY’s guideline, after delivery in a public or accredited private health facility, eligible women receive Rs 600 in urban areas and Rs 700 in rural areas. In ten High Focus- Non North Eastern (NE) states (Uttar Pradesh, Uttarakhand, Bihar, Jharkhand, Madhya Pradesh, Chhattisgarh, Himachal Pradesh, Rajasthan, Orissa, and Jammu & Kashmir) all pregnant women are eligible, and benefits are paid regardless of whether they deliver in a government or in a private accredited institution, and regardless of birth order. Benefits for institutional delivery are Rs. 1,400 in rural areas and Rs. 1,000 in urban areas. In Non High Focus states, women are eligible for the cash benefit only for their first two live births and only if they had a below poverty line (BPL) card issued by the government or if they were from a scheduled caste or tribe. Pregnant women can also receive cash assistance for transport to the nearest government health facility for delivery. Each state determines the amount of assistance, but the minimum is Rs. 250. It is paid to pregnant women on arrival and registration at the facility. Women who deliver at home are still eligible for a cash payment to cover the expenses associated with delivery, but only if they are 19 years of age and older, belong to BPL household and gave birth to their first or second child. Such mothers are entitled to Rs. 500 per delivery. JSY is being implemented through Accredited Social Health Activists (ASHAs), who identify pregnant women and help them to get to a health facility. ASHAs receive payments of Rs. 200 in urban areas and Rs. 600 in rural areas per in-facility delivery assisted by them in high focus states. *[14,15,17].

Although a small number of micro studies [18-22] have provided estimates of OOPE to family for delivery care, these estimates were confined to small geographic areas in India. We used a nationally representative cross-sectional dataset [District Level Household and Facility Survey-Phase 3 (DLHS-3)], to provide robust estimates of OOPE to family of delivery care for all the states and union territories in India, except for Nagaland, as it was not covered under DLHS-3. Specific objectives of our study are:

1. To estimate the average OOPE for women/families according to the type (normal/caesarean-section) and place (home/government hospital/private hospital) of delivery, in the states/union territories of India;

2. To examine inter-state variations in percent JSY beneficiaries and percent families who had to borrow money/sell property to meet the delivery expenses for normal and caesarean-section deliveries;

3. To outline how average OOPEs, percent JSY beneficiaries and percent families borrowing vary for normal/caesarean-section deliveries according to socio-demographic profiling of families in India.

## Methods

The DLHS-3 collected data on OOPE to family on delivery care from ever married women who had a live/still birth between January 2004 and December 2008. However, we confined our analysis to births/deliveries between January 2007 and December 2008, as state-wise implementation of the JSY programme was highly variable during previous years [14].

We have adopted the DLHS-3 definition for ‘type of delivery’ and ‘place of delivery’ [23]. A delivery not requiring intervention in the form of an operation/use of forceps/ cut and stitches was termed ‘normal vaginal delivery’; an operation was termed ‘caesarean-section’ (‘c-section’); and the use of forceps/cut/ stitches was termed ‘instrument/assisted’ delivery. A delivery in a public institution [Government hospital, dispensary, urban health centre/post/family welfare centre, community health centre/rural hospital, primary health centre, sub centre, Ayurveda, Yoga, Unani, Siddha, & Homeopathy (AYUSH) hospital/clinic] was classified as ‘public institution delivery’. A delivery in a private hospital/clinic or private AYUSH hospital/clinic, was classified ‘private institution delivery’. A delivery in a woman’s or her parents’ home was classified as ‘home delivery’, and a delivery occurring at a Non-governmental organisation (NGO)/Trust hospital/clinic, en route to the hospital, work place, other places was classified as ‘other place’.

The OOPE incurred by family on delivery care, percent families who had borrowed money/sold property for meeting delivery care expenses, percent JSY beneficiary families/women are the main outcome measures of this study. Expenditure incurred by the woman/family on transportation was obtained only for institutional deliveries. If there was no expenditure on transportation it was coded as ‘0’, else the actual expenditure was coded, up to a maximum of Rs. 89,999 ^a^. Delivery care expenditures (irrespective of place and type) include: antenatal care (ANC), delivery, and medicines during the period [23]. If no expenditure was incurred for delivery, it was coded as Rs. ‘0’, otherwise the actual expenditure was coded up to a maximum of Rs. 99,996 ^b^. By adding expenditures on transportation and delivery care we have computed a new variable, ‘out-of-pocket expenditure (OOPE) of a delivery’.

States and union territories of India were grouped according to the National Rural Health Mission (NRHM) classification [24], as JSY compensation policies mainly vary according to this classification [14-16]:

• 10 High Focus - Non North Eastern (NE) states;

• 7 High Focus – NE states;

• 11 Non High Focus – Large states; and

• 6 Non High Focus – Small States & Union Territories (UT).

We also measured variations in OOPE on normal/c-section delivery according to the following socio-demographic characteristics:

• Caste (scheduled caste, scheduled tribe, other backward caste, others);

• Maternal education (no education, 1–5 years, 6–11 years, and 12 years or more);

• Quintiles of household wealth index (poorest, second, middle, fourth, richest);

• Location of residence (rural, urban);

• Pregnant women’s interaction with health worker [registered the pregnancy and got advice (at least once) on institutional delivery] (yes, no); and

• Got full ANC (yes, no).

### Statistical analysis

The OOPE to family of delivery care was analyzed by estimating mean & standard deviation (SD) and median & inter-quartile range (IQR) values, because OOPE on delivery care data were heavily skewed. Chi-square and one-way analysis of variance (ANOVA) tests were used to test significance of difference between proportions and means respectively. We applied weights for the state in entire analysis. Analysis was undertaken in SPSS-19.

## Results

The response rate of women who had a live/still birth between January 2007 and December 2008 in DLHS-3 was 93% (N=92,563). Out of these women, data on OOPE for delivery care were available for 83,510 (90.2%), and information on OOPE and type of delivery and place of delivery was available for 83,493 (90.2%). The mean OOPE to family, only on ANC and delivery care for all births in India in 2007/08 was Rs. 2,037 (SD=4,509) and median of Rs. 500 (IQR=150-2,000). Mean expenditure exclusively on transportation for the 36,524 (39%) women who had an institutional delivery was Rs. 322 (SD=893), median of Rs. 150 (IQR=50-400). Mean (total) OOPE to family for maternity/delivery care (transportation + ANC+ delivery expenditure) was Rs. 2,169 (SD=4,647) with a median of Rs. 600 (IQR=200-2,000).

### Flow chart

Summary profile of delivery care in India by type (normal/caesarean-section) and location private institution/private institution/home), in 2007–08.

**Figure 1:**
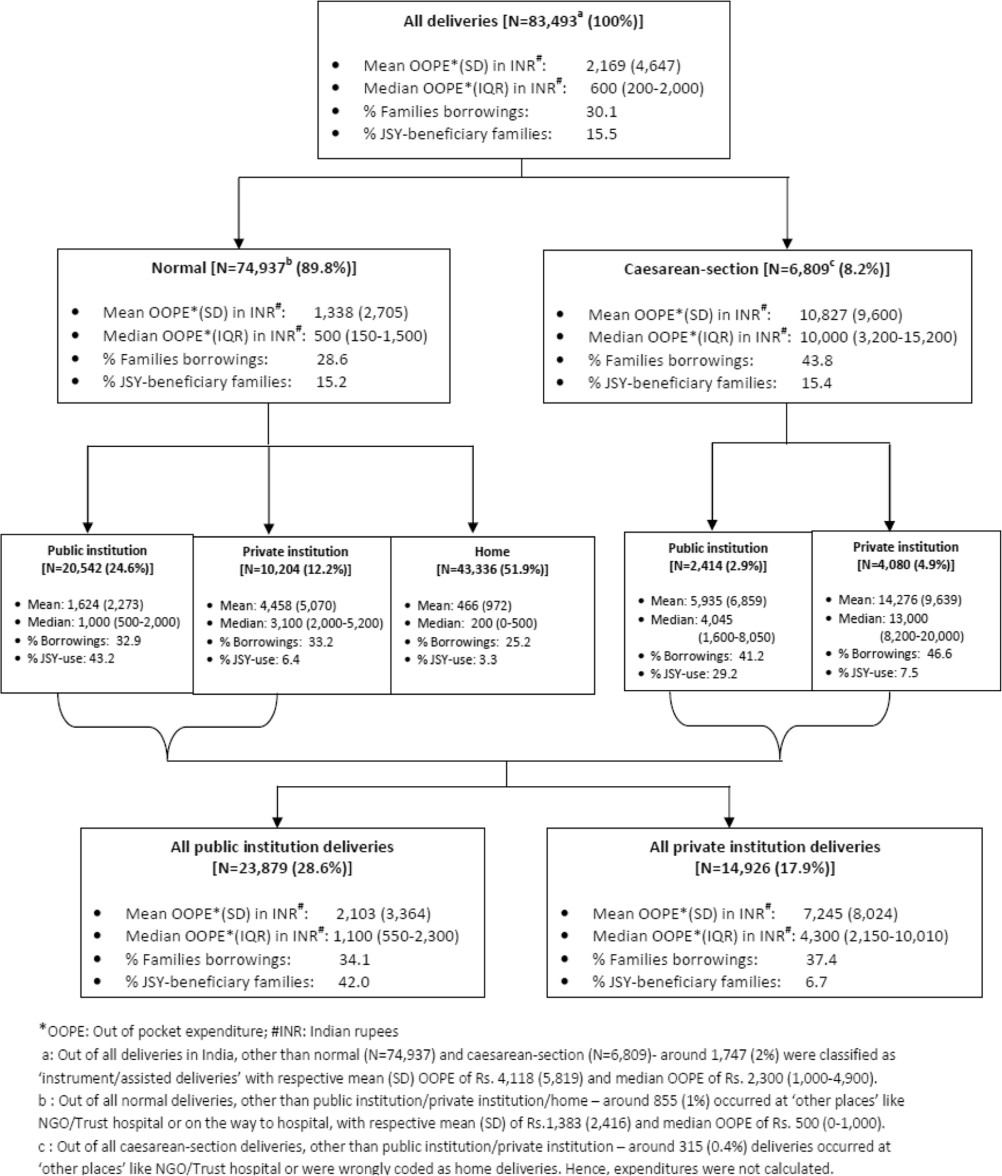
Summary profile of delivery care in India by type (normal/caesarean-section) and location (public institution/private institution/home) in 2007-08.

Figure 1 provides a summary profile of OOPEs associated with delivery care in India according to type and place of delivery. Of all the deliveries in India in 2007–08, 90% were classified as ‘normal’ , 8% as ‘c-section’  and 2% as ‘instrument/assisted’. The breakdown of the 90% normal deliveries by location of delivery was as follows: home (52%); government hospital (25%); private hospital (12%); and others/NGOs (1%). The breakdown of the 8% of c-sections was as follows: private institution (5%); public institution (3%); and others (0.4%). The mean OOPE associated with a c-section birth was eight times that for a normal delivery, and high expenditures associated with these c-sections forced almost one-in-two women/families to borrow money. Mean OOPE of a normal delivery in public institution (Rs. 1,624) was three times that for a home delivery (Rs. 466), while a normal delivery in a private institution (Rs. 4,458) was three times that occurring in a public institution. One in every four women/families who had a normal delivery at home borrowed money, even though mean expenditure was only Rs. 466. One in every three women who had a normal delivery in public/private institution borrowed money. The JSY programme reach was mainly confined to public institution deliveries (43%) with almost negligible reach to private institution (6%) or home (3%) deliveries.

### State level variations in OOPE, borrowings and JSY use for normal deliveries at public and private institutions and at home

**Figure 2:**
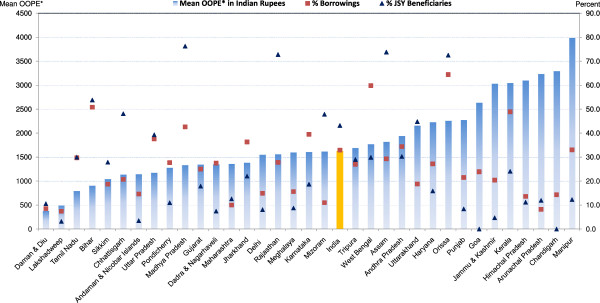
Mean OOPE* to family on normal delivery at public facility, percent borrowings and percent JSY-beneficiaries by state. OOPE*: Out of Pocket Expenditure.

Figure 2 presents mean OOPE, percent borrowings and percent JSY beneficiaries of a normal delivery in a public institution by state/UT (see Tables 1 and 2 for more detailed information). With large interstate variations, mean OOPE of a normal delivery in a public institution was least expensive (Rs. 381) in Daman & Diu and most expensive in Manipur (Rs. 3,984), with a national average of Rs. 1,624. In only nine out of 34 states/UTs, median OOPE was less than the JSY compensation amount of Rs. 700 (Table 1). Mean OOPE is not the sole determinant of families having to borrow money. For example, despite high mean OOPE (Rs. 3,230) in Arunachal Pradesh, only 8 percent families opted for borrowing, while despite a low mean OOPE (Rs. 1,769) in West Bengal, a large proportion (60%) of families opted to borrowings. There were considerable state-wise variations in percent JSY beneficiaries even among the ten high focus- non NE states (76% in Madhya Pradesh and 5% in Jammu & Kashmir), when 100% these women are technically eligible to receive JSY benefit. Among the high focus - NE states, Assam did well in terms of JSY outreach followed by Mizoram. In non high focus states/UTs, JSY use was generally low.

**Table 1:** **Mean and median out**-**of**-**pocket**-**expenditure (OOPE) of normal vaginal deliveries by location and state/UT**

**STATE**	**% of all deliveries**	**Mean OOPE in INR (SD)**				**Median OOPE in INR (IQR)**			
		**Public**	**Private**	**Home**	**All normal**	**Public**	**Private**	**Home**	**All normal**
**High Focus - Non NE**									
Jammu & Kashmir	82.4	3,028 (2443)	5,090 (4468)	760 (1112)	1,920 (2441)	2,500 (1300–4000)	3,950 (2226–6164)	400 (0–1000)	1,000 (300–2800)
Himachal Pradesh	84.1	3,093 (2785)	6,768 (6471)	630 (920)	1,752 (2823)	2,400 (1200–4000)	5,316 (3916–8347)	500 (0–800)	600 (200–200)
Uttarakhand	93.0	2,156 (2060)	5,472 (3702)	505 (630)	1,234 (2038)	1,500 (1000–3000)	5,071 (3000–7000)	500 (100–600)	500 (200–1053)
Rajasthan	95.2	1,557 (1413)	3,522 (2981)	504 (1945)	1,209 (2038)	1,200 (750–1900)	2,800 (1742–4522)	300 (100–500)	700 (250–1500)
Uttar Pradesh	94.1	1,172 (1707)	3,623 (5370)	320 (633)	811 (2261)	750 (500–1250)	2,200 (1200–4100)	184 (18–400)	251 (51–700)
Bihar	93.9	904 (1188)	2,961 (3020)	615 (1103)	888 (1545)	600 (400–1033)	2,062 (1200–3500)	400 (200–600)	500 (200–1000)
Jharkhand	94.4	1,382 (2036)	3,742 (3157)	440 (605)	754 (1462)	800 (463–1620)	3,005 (1900–5000)	300 (200–500)	300 (200–600)
Orissa	82.8	2,260 (2408)	4,261 (3375)	316 (801)	1,229 (2069)	1,700 (1050–2531)	3,300 (1587–5936)	0 (0–400)	500 (0–1650)
Chhattisgarh	94.6	1,135 (1783)	4,251 (3652)	179 (323)	480 (1351)	670 (320–1100)	3,500 (1929–5712)	0 (0–300)	60 (0–500)
Madhya Pradesh	94.6	1,331 (1954)	4,965 (5777)	574 (962)	1,217 (2284)	800 (500–1500)	3,254 (2000–5500)	300 (20–700)	600 (200–1300)
**High Focus - NE**									
Sikkim	83.7	1,043 (710)	7,700* (2105)	148 (298)	573 (874)	977 (500–1500)	7,700* (6200–9200)	0 (0–100)	300 (0–900)
Arunachal Pradesh	94.7	3,230 (4697)	9,208 (11317)	518 (1319)	1,844 (3942)	2,000 (880–4014)	7,152 (2200–10197)	0 (0–500)	500 (90–2070)
Manipur	90.8	3,984 (3731)	7,617 (4471)	419 (712)	1,870 (3209)	3,300 (2200–5029)	7,500 (4397–10000)	200 (0–500)	500 (100–2500)
Mizoram	94.6	1,616 (2276)	6,435 (4180)	350 (492)	1,289 (2242)	1,050 (650–1700)	5,300 (3330–9521)	200 (0–500)	700 (200–1500)
Tripura	91.3	1,688 (2074)	3,824 (3435)	112 (251)	700 (1530)	1,000 (600–2000)	3,056 (822–7841)	0 (0–150)	150 (0–800)
Meghalaya	89.7	1,600 (2373)	5,024 (5778)	225 (596)	664 (1947)	900 (400–2000)	2,832 (1469–5967)	0 (0–300)	150 (0–500)
Assam	91.3	1,818 (3362)	6,280 (5593)	194 (477)	918 (2517)	1,200 (700–2100)	5,000 (2400–8211)	0 (0–250)	220 (0–1000)
**Non High Focus - Large**									
Punjab	83.9	2,272 (1886)	3,517 (2237)	974 (876)	2,172 (2044)	2,000 (1030–3000)	3,050 (2100–4400)	800 (500–1100)	1,650 (800–3000)
Haryana	86.3	2,228 (3576)	5,902 (5996)	844 (1508)	2,415 (4186)	1,000 (400–2800)	4,839 (3000–7000)	500 (200–1000)	900 (250–3015)
Delhi	81.1	1,547 (2898)	10,372 (9916)	945 (1570)	3,491 (6618)	600 (150–1632)	8,000 (5000–12184)	600 (300–1100)	1,000 (400–3623)
Gujarat	90.7	1,344 (1969)	3,267 (3956)	298 (529)	1,511 (2796)	800 (250–1852)	2,200 (1446–3877)	150 (0–380)	560 (101–2000)
Maharashtra	89.2	1,356 (1878)	4,345 (5569)	641 (1090)	2,043 (3743)	700 (332–1700)	3,052 (2000–5050)	300 (0–1000)	1,000 (300–2500)
Andhra Pradesh	71.4	1,937 (2280)	5,071 (5502)	1,070 (2014)	2,589 (3917)	1,100 (600–2150)	3,472 (2100–5482)	500 (100–1500)	1,500 (500–3100)
Karnataka	83.9	1,604 (1791)	4,667 (4941)	756 (1174)	2,190 (3383)	1,100 (600–2050)	3,300 (2050–5488)	500 (50–1000)	1,100 (500–3000)
Goa	71.3	2,630 (3193)	7,685 (4848)	319 (453)	4,859 (4797)	2,000 (300–3600)	6,719 (4365–10000)	67 (0–780)	4,037 (1182–7026)
Kerala	65.9	3,040 (2676)	6,624 (5282)	1,394 (2106)	5,332 (4843)	2,100 (1100–4210)	5,160 (4000–8000)	150 (75–3478)	4,800 (2500–6377)
Tamil Nadu	73.7	792 (1142)	4,926 (4808)	102 (365)	2,062 (3442)	418 (50–1030)	3,910 (2549–5831)	0 (0–0)	850 (100–3000)
West Bengal	85.6	1,769 (2163)	5,082 (4970)	568 (863)	1,197 (2050)	1,096 (568–2100)	4,042 (2010–6172)	300 (100–600)	500 (200–1300)
**Non High Focus - Small states & UTs**									
Chandigarh	87.4	3,288 (3613)	6,748 (5767)	943 (500)	3,293 (4226)	2,550 (2000–3175)	5,000 (4000–8200)	900 (500–1200)	2,000 (750–4080)
Daman & Diu	83.3	381 (780)	4,543 (2804)	490 (666)	1,908 (2620)	100 (1–287)	4,100 (2500–5316)	207 (100–500)	500 (100–3000)
Dadra & Nagarhaveli	95.3	1,351 (1662)	6,063 (2960)	616 (1013)	1,536 (2440)	545 (50–2127)	7,028 (4191–7447)	200 (100–700)	300 (100–2000)
Lakshadweep	76.7	494 (1645)	21,750 (14947)	233 (331)	1,570 (5855)	50 (20–60)	17,000 (10375–37875)	0 (0–500)	50 (0–500)
Pondicherry	66.4	1,276 (1707)	6,500 (3478)	75* (103)	2,565 (3207)	563 (150–1700)	5,255 (4100–8670)	75* (0–75)	1091 (311–3858)
Andaman & Nicobar Islands	86.9	1,143 (3028)	6,463 (5617)	909 (1965)	1,191 (2929)	100 (0–1000)	9,500 (0–9992)	0 (0–899)	99 (0–1000)
**India - Total**	**89.7**	**1,624 (2273)**	**4,458 (5070)**	**466 (972)**	**1,338 (2705)**	**1,000 (500–2000)**	**3,100 (2000–5200)**	**200 (0–500)**	**500 (150–1500)**

*: Estimates based on less than 5 cases; INR: Indian National Rupees; SD: Standard Deviation; Public: Public institution; Private: Private institution; IQR: Inter-quartile range; NE: North Eastern; UT: Union Territory.

**Table 2:** Percent borrowings and percent JSY beneficiaries by delivery type, location and state/UT

	**% Borrowings**							**% JSY beneficiaries**						
**STATE**	**Normal**				**Caesarian**			**Normal**				**Caesarian**		
	**Public**	**Private**	**Home**	**All normal**	**Public**	**Private**	**All c-section**	**Public**	**Private**	**Home**	**All normal**	**Public**	**Private**	**All c-section**
**High Focus - Non NE**														
Jammu & Kashmir	20.4	34.2	12.8	17.4	25.9	26.8	25.7	4.7	1.8	0.8	2.6	4.9	3.6	4.5
Himachal Pradesh	13.6	23.6	9.9	12.0	25.9	41.3	32.0	11.2	3.6	2.3	5.4	9.9	1.6	6.2
Uttarakhand	18.8	26.9	9.9	13.2	34.1	50.8	43.3	44.8	1.4	1.9	10.9	31.7	1.7	13.5
Rajasthan	27.8	29.8	15.4	22.1	33.7	42.4	36.6	72.8	4.4	1.5	32.3	55.1	5.4	28.3
Uttar Pradesh	37.6	44.2	27.4	30.8	45.3	56.1	51.8	39.3	2.4	1.2	6.2	30.0	3.0	8.0
Bihar	50.8	52.8	47.9	48.9	61.3	68.6	65.1	53.8	2.7	1.3	13.5	33.9	4.1	9.3
Jharkhand	36.3	32.8	30.3	30.9	53.6	54.0	53.4	22.1	2.3	1.6	3.8	17.9	5.1	6.9
Orissa	64.4	49.5	29.6	44.5	71.9	59.2	61.3	72.5	17.5	9.5	35.5	66.1	9.9	43.1
Chhattisgarh	20.7	31.8	10.0	12.4	52.6	46.4	44.8	48.1	6.8	4.0	10.5	34.2	3.6	14.3
Madhya Pradesh	42.6	31.7	31.8	37.6	59.7	44.4	51.4	76.3	9.8	4.1	42.9	70.6	3.7	34.4
**High Focus - NE**														
Sikkim	18.7	25.0*	11.4	14.8	21.4	15.4	20.0	27.9	0.0*	19.4	23.2	26.2	7.7	21.8
Arunachal Pradesh	8.2	0.0	1.6	4.6	9.6	12.5	9.7	12.0	5.3	1.4	6.1	5.8	25.0	8.1
Manipur	33.0	36.2	8.2	17.1	40.0	40.8	39.6	12.3	2.6	3.3	5.7	13.8	6.6	9.7
Mizoram	11.0	13.2	3.9	7.9	27.1	9.1	22.2	47.9	47.4	5.0	29.0	41.7	9.1	34.9
Tripura	27.0	42.9	7.3	15.4	39.4	18.2	33.3	29.0	0.0	2.0	12.1	18.2	0.0	13.3
Meghalaya	15.6	10.0	8.9	10.4	23.5	18.2	15.4	8.8	2.0	1.3	2.7	5.9	8.3	3.8
Assam	29.3	26.0	11.9	18.1	41.0	28.8	34.4	73.8	14.2	3.0	26.9	59.6	3.9	33.9
**Non High Focus - Large**														
Punjab	21.5	25.4	36.2	29.4	38.1	44.9	43.6	8.4	1.0	1.4	2.5	10.6	1.1	3.4
Haryana	27.2	34.1	30.5	31.0	34.2	49.8	46.3	15.9	3.0	3.7	5.4	11.8	2.0	4.2
Delhi	14.9	19.3	27.7	20.8	18.8	25.9	23.2	8.1	1.4	0.3	3.5	4.7	1.2	2.6
Gujarat	25.0	30.8	18.4	24.2	31.6	38.2	36.8	17.9	10.6	6.2	10.4	17.5	5.3	7.9
Maharashtra	10.0	13.4	6.8	9.9	15.3	18.4	17.0	12.6	2.9	11.3	9.0	15.3	3.0	6.6
Andhra Pradesh	34.4	47.7	30.9	37.3	51.6	60.3	58.2	30.3	20.3	6.0	18.6	41.4	18.1	23.1
Karnataka	39.5	34.6	26.3	33.3	50.9	42.2	45.7	18.7	10.1	9.7	13.1	19.6	14.3	16.6
Goa	23.9	22.5	33.3	23.9	50.0	7.4	18.9	0.0	0.0	0.0	0.0	0.0	3.7	2.7
Kerala	48.9	31.7	25.0	38.1	62.7	45.8	51.2	24.1	7.2	12.5	13.5	26.1	4.8	11.6
Tamil Nadu	29.7	41.4	8.0	32.0	44.2	59.4	53.9	30.0	22.7	23.9	27.3	43.2	23.7	30.2
West Bengal	59.8	41.2	47.1	51.5	60.8	44.3	52.5	29.9	10.4	15.1	20.1	24.6	9.2	16.3
**Non High Focus - Small States & UTs**														
Chandigarh	14.3	16.7	30.4	19.7	60.0	0.0*	37.5	0.0	0.0	0.0	0.0	0.0	0.0*	0.0
Daman & Diu	8.5	23.9	11.0	15.3	25.0	44.7	41.3	10.6	2.3	0.0	2.8	0.0	2.6	2.2
Dadra & Nagarhaveli	27.5	26.1	35.1	32.3	0.0*	20.0	12.5	7.5	8.7	0.0	3.1	0.0	0.0	0.0
Lakshadweep	7.4	0.0	0.0	5.7	0.0	23.8	13.5	3.2	0.0	0.0	2.5	6.3	0.0	2.7
Pondicherry	27.7	20.0	0.0*	25.6	30.0	18.0	24.5	11.0	1.7	0.0	8.2	16.7	0.0	9.1
Andaman & Nicobar Islands	14.6	0.0	10.9	13.1	16.7	66.7	20.7	3.5	0.0	1.8	2.9	4.2	0.0	3.4
**India - Total**	**32.9**	**33.2**	**25.2**	**28.6**	**41.2**	**46.6**	**43.8**	**43.2**	**6.4**	**3.3**	**15.2**	**29.2**	**7.5**	**15.4**

*: Estimates based on less than 5 cases; Borrowings: Households who borrowed money/sold property for meeting delivery expenses; Public: Public institution; Private: Private institution; NE: North Eastern; UT: Union Territory.

**Figure 3:**
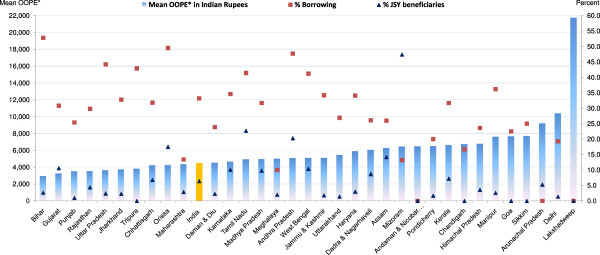
Mean OOPE* to family on normal delivery at private facility, percent borrowings and percent JSY-beneficiaries by state. OOPE*: Out of Pocket Expenditure.

Figure 3 presents mean OOPE, percent borrowings and percent JSY beneficiaries for a normal delivery in a private institution by state/UT (see Tables 1 and 2 for additional data). Excluding Bihar, Lakshadweep, Delhi and Arunachal Pradesh - mean OOPE for a normal delivery in the remaining states ranged from Rs. 3,000-8,000. Irrespective of mean OOPE, these deliveries were generally associated with higher borrowings and fewer JSY beneficiaries. At national level, only 6% of these deliveries received JSY benefit. Borrowings for these deliveries were high in Bihar, Orissa and Andhra Pradesh, while percent borrowings were lower in Maharashtra, Meghalaya and Mizoram. Irrespective of NRHM classification of states/ UTs, JSY reach to deliveries in private institutions was generally poor across all the states, excluding Tamil Nadu, Andhra Pradesh and Mizoram, where more than 20% received JSY benefit.

**Figure 4:**
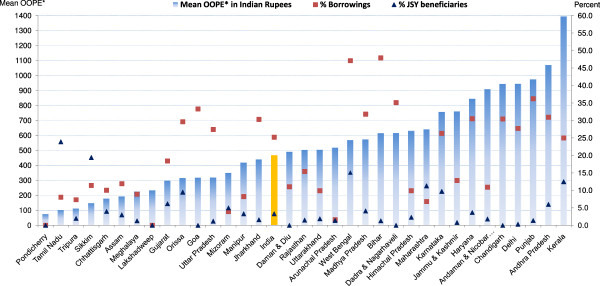
Mean OOPE* to family on normal delivery at home, percent borrowings and percent JSY-beneficiaries by state. OOPE*: Out of Pocket Expenditure.

Figure 4 presents mean OOPE, percent borrowings and percent JSY beneficiaries for a normal delivery at home by state/UT (see Tables 1 and 2 for additional data). Mean OOPE for a normal delivery at home was Rs. 466, with one-fourth of women/families requiring to borrow money, while a negligible (3%) proportion of them received the JSY benefit. Mean OOPE of a home delivery across the states/UTs may broadly be divided into three broad groups: less than Rs. 500 in 16 states; between Rs. 500–1,000 in 16; and more than Rs. 1000 in two states. High mean OOPE of these deliveries were generally associated with high borrowings and poor JSY outreach (less than 10% in 29 of the 34 states/UTs).

### State level variations in OOPE, borrowings and JSY use for c-section deliveries at public and private institutions

**Figure 5:**
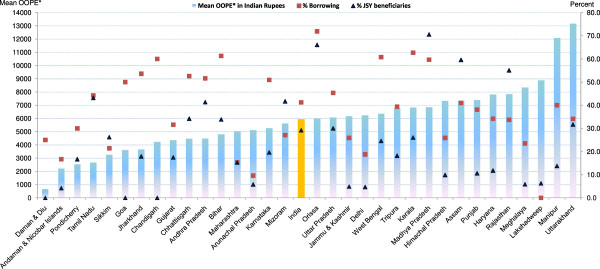
Mean OOPE* to family on caesarean-section at public facility, percent borrowings and percent JSY-beneficiaries by state. OOPE*: Out of Pocket Expenditure.

**Figure 6:**
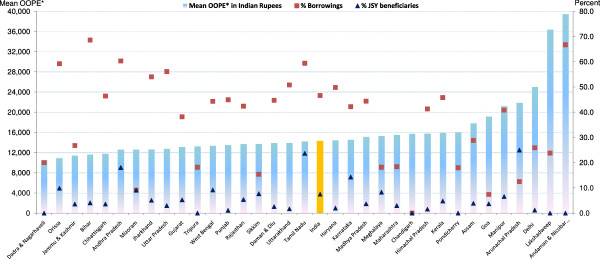
Mean OOPE* to family on caesarean-section at private facility, percent borrowings and percent JSY-beneficiaries by state. OOPE*: Out of Pocket Expenditure.

Data on mean OOPE, percent borrowings and percent JSY beneficiaries for c-section deliveries at public and private institutions by state/UT is presented in Figures 5 and 6 and Tables 2 and 3. Mean OOPE for a c-section in a public institution was Rs. 5,935, ranging from Rs. 678 in Daman & Diu to Rs. 13,165 in Uttarakhand. These deliveries, despite occurring in public institutions, have forced more than two-fifths (41%) of families to borrow money, with less than one-third (29%) receiving the JSY-benefit. Percent families borrowings for these public institution c-section deliveries were 60% or more in the states of: Chandigarh, Bihar, Orissa, West Bengal and Kerala; while percentage of families benefitting through JSY program was less than 10% in Daman & Diu, Andaman & Nicobar Islands, Goa, Chandigarh, Arunachal Pradesh, Jammu & Kashmir, Delhi, Himachal Pradesh, Meghalaya, Dadra & Nagarhaveli and Lakshadweep. Mean OOPE for a c-section in a private institution was Rs. 14,276, ranging from Rs. 10,554 in Dadra & Nagarhaveli to Rs. 39,424 in Andaman & Nicobar Islands. For these c-sections almost half (47%) of the families had to opt for borrowings with state/UT wise variation ranging from 7% to 69% (Tables 2 and 3). Use of the JSY programme among women having a c-section in a private institution was 8% nationally, ranging from 0% to 25%. Irrespective of NRHM classification of states/UTs, these deliveries were generally associated with higher borrowings and fewer JSY benefits.

**Table 3:** **Mean and median out**-**of**-**pocket**-**expenditure (OOPE) of caesarean-section deliveries by location and state/UT**

**STATE**	**% all deliveries**	**Mean cost in INR (SD)**			**Median cost in INR (IQR)**		
		**Public**	**Private**	**All c-section**	**Public**	**Private**	**All c-section**
**High Focus - Non NE**							
Jammu & Kashmir	14.9	6,182 (4879)	11,428 (6687)	6,876 (5621)	5,010 (2446–8930)	11,000 (5794–16000)	5,300 (2497–10078)
Himachal Pradesh	11.4	7,330 (6677)	15,791 (10174)	10,935 (9436)	5,500 (3011–10084)	15,326 (7709–20407)	8,000 (3500–15790)
Uttarakhand	5.1	13,165 (18021)	13,916 (10037)	13,811 (13406)	8115 (3000–17308)	15,500 (5000–20395)	12,109 (3867–20143)
Rajasthan	3.7	7,843 (10375)	13,718 (9264)	10,336 (10224)	5021 (2000–9060)	11,683 (6495–20000)	8,144 (2634–15300)
Uttar Pradesh	4.6	6,097 (6478)	12,767 (10377)	10,614 (10039)	3,298 (1500–9828)	10,500 (4089–17392)	9,154 (2500–15500)
Bihar	3.7	4,810 (5446)	11,644 (8156)	9,351 (8257)	2,881 (813–7189)	10,256 (5000–15235)	8,100 (2097–15000)
Jharkhand	3.9	3,670 (3250)	12,670 (8481)	11,069 (8552)	2,600 (1344–5311)	11,837 (7027–17000)	10,034 (3956–16000)
Orissa	10.1	5,990 (6010)	10,949 (7412)	6,286 (6753)	3,300 (1700–10011)	10,020 (4164–15414)	3,273 (1277–10200)
Chhattisgarh	4.6	4,477 (3533)	11,790 (6121)	7,870 (6520)	2,745 (1533–7070)	10,702 (8004–15194)	6,869 (2092–11649)
Madhya Pradesh	3.7	6,855 (7316)	15,136 (9221)	11,152 (9368)	4,468 (1300–10074)	15,014 (10020–20060)	10,050 (2800–16027)
**High Focus - NE**							
Sikkim	10.2	3,261 (1957)	13,748 (10035)	5,989 (6997)	2,657 (2026–4028)	10,752 (7006–17928)	3,172 (2108–6827)
Arunachal Pradesh	5.0	5,123 (6478)	21,905 (47483)	8,237 (20947)	3,100 (1015–8000)	5,457 (2915–8834)	3,381 (1276–8000)
Manipur	8.2	12,086 (6710)	21,158 (8074)	16,685 (8883)	10,702 (8083–15203)	20,588 (16820–25825)	15,560 (10187–20800)
Mizoram	4.8	5,615 (3347)	12,620 (8148)	7,093 (5359)	5,000 (3433–7217)	12,674 (6321–15100)	5,322 (4000–10080)
Tripura	6.3	6,774 (4252)	13,265 (3156)	8,036 (4957)	7,074 (3822–10009)	12,201 (11104–14835)	7,927 (4145–12005)
Meghalaya	3.2	8,346 (14016)	15,303 (8279)	7,489 (10636)	3,478 (1362–10753)	14,478 (8546–22772)	2,456 (472–11000)
Assam	5.9	7,342 (7596)	17,825 (11868)	10,445 (10755)	5,652 (2300–10119)	18,106 (7500–25000)	7,000 (2215–16272)
**Non High Focus - Large**							
Punjab	13.6	7,383 (5067)	13,525 (7028)	12,029 (7117)	6,050 (3500–10168)	12,962 (10000–17144)	11,008 (6050–15487)
Haryana	10.5	7,807 (7597)	14,450 (10765)	13,017 (10515)	6,429 (1880–10438)	15,000 (5282–20100)	12,374 (4514–20000)
Delhi	14.2	6,246 (11543)	25,028 (16415)	17,525 (17233)	3,050 (1044–5932)	21,395 (13015–35000)	13,016 (3050–25762)
Gujarat	7.4	4,367 (7818)	13,099 (9232)	11,220 (9539)	2,330 (200–5235)	12,020 (8120–15906)	10,200 (3300–15066)
Maharashtra	10	5,042 (5012)	15,509 (8498)	12,175 (8991)	4,027 (1500–6577)	15,052 (10050–20020)	11,000 (5018–17146)
Andhra Pradesh	27.6	4,499 (4400)	12,602 (6888)	10,700 (7269)	3,158 (1500–5100)	10,300 (9099–15200)	10,050 (5100–15050)
Karnataka	14.0	5,271 (5743)	14,572 (9519)	10,903 (9400)	4,008 (2000–6470)	13,279 (10005–18100)	10,025 (3524–15100)
Goa	28.7	3,617 (3350)	19,158 (16120)	15,034 (15494)	4,000 (416–5263)	15,115 (14175–21862)	15,000 (4818–17745)
Kerala	34.0	6,830 (7551)	15,933 (10055)	13,294 (10234)	5,100 (3253–7152)	14,029 (10274–19000)	12,005 (6138–16000)
Tamil Nadu	24.9	2,674 (4619)	14,239 (6819)	10,295 (8207)	1,249 (266–3082)	14,963 (10025–17143)	10,050 (2380–15100)
West Bengal	11.4	6,367 (5118)	13,357 (7800)	10,131 (7516)	5,400 (3066–8072)	11,000 (8150–16035)	8,400 (5054–12733)
**Non High Focus - Small states & UTs**							
Chandigarh	9.2	4,233 (2246)	15,750* (12078)	9,169 (9436)	3,790 (2363–6545)	20,100* (2100–20100)	5,030 (2300–20100)
Daman & Diu	15.4	678 (1094)	13,893 (6143)	11,463 (7594)	77 (0–1118)	15,000 (8546–20000)	12,055 (5011–16611)
Dadra & Nagarhaveli	4.7	--	10,554 (6612)	10,554 (6612)	--	10,204 (5244–16257)	10,204 (5244–16257)
Lakshadweep	23.3	8,876 (19767)	36,345 (17837)	25,323 (22824)	1,014 (30–7668)	29,604 (24070–56986)	24,521 (1500–49561)
Pondicherry	33.3	2,542 (3102)	16,073 (9096)	8,433 (9295)	1,100 (550–3000)	15,000 (10151–20056)	5,890 (1013–13318)
Andaman & Nicobar Islands	12.2	2,228 (3059)	39,424 (31749)	5,514 (13279)	565 (50–5000)	39,775 (17500–39775)	797 (39–5042)
**India - Total**	**8.2**	**5,935 (6859)**	**14,276 (9639)**	**10,827 (9600)**	**4,045 (1600–8050)**	**13,000 (8200–20000)**	**10,000 (3200–15200)**

*: Estimates based on less than 5 cases; INR: Indian National Rupees; SD: Standard Deviation; Public: Public institution; Private: Private institution; IQR: Inter-quartile range; NE: North Eastern states; UT: Union territory.

### Socio-demographic variations in OOPE, borrowings and JSY use for all normal/c-section deliveries in India

**Figure 7:**
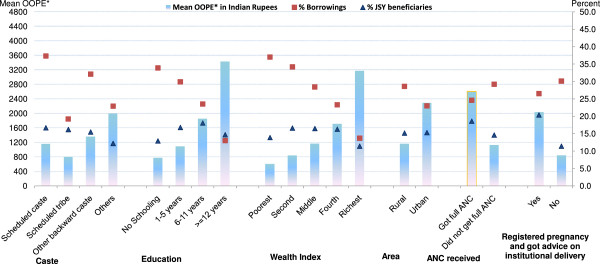
Mean OOPE* to family on normal delivery, percent borrowings and percent JSY-beneficiaries by socio-demographic profiling of women, India. OOPE*: Out of Pocket Expenditure.

**Figure 8:**
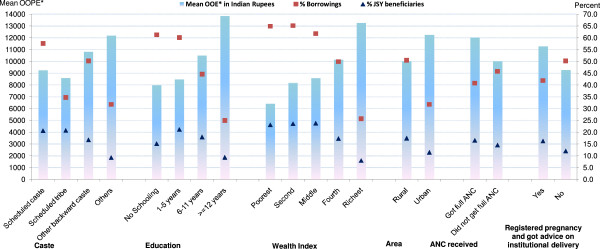
Mean OOPE* to family on caesarean-section delivery, percent borrowings and percent JSY-beneficiaries by socio-demographic profiling of women, India. OOPE*: Out of Pocket Expenditure.

Variations in mean OOPE, percent borrowings and percent JSY beneficiaries – according to socio-demographic profiling of all normal and c-section deliveries in India are presented in Figures 7 and 8 (see Tables 4 and 5 for additional data). Mean OOPE of a normal delivery was significantly higher for other caste: Rs. 1,996; ≥12 years educated: Rs. 3,429; and richest: Rs. 3,170 women, as compared to their scheduled tribe Rs. 805; illiterate Rs. 775; and poorest Rs. 605 counterparts. Higher literacy and wealth was associated with fewer borrowings but had no influence on use of the JSY-benefit. Mean OOPE of a normal delivery in urban areas (Rs. 2,290) was around two times the rural area (Rs. 1,163). Proportion borrowing and JSY reach did not differ significantly by rural/urban differentials. Mean OOPE of a normal delivery was more than double in those who had full ANC or who interacted with a health worker during pregnancy as compared to their respective group counterparts. JSY reach and proportion borrowing did not differ significantly according to ANC use and women’s interaction with health worker (Table 4). Excluding education and wealth index, in the remaining socio-demographic groups, variations in mean OOPE and % borrowing were less evident among the c-section deliveries (Figure 8), as compared to normal deliveries, in India. The OOPE on c-sections did not differ significantly according to type of area (rural/urban), receiving full ANC care (yes/no) and pregnant woman’s interaction with health worker (yes/no) (Table 5). For poor and illiterate women, expenditures on c-sections were beyond their capacity to pay resulted in significantly more borrowings.

**Table 4:** **Mean & median out**-**of**-**pocket**-**expenditure (OOPE) of normal deliveries, percent borrowings and percent JSY-beneficiaries according to socio economic profile of women**

**Socio-demographic characteristics**	**Public institution**				**Private institution**				**Home**				**Total Women**
	**Mean OOPE in INR (SD)**	**Median OOPE in INR (IQR)**	**% Borrowing**	**% JSY beneficiaries**	**Mean OOPE in INR (SD)**	**Median OOPE in INR (IQR)**	**% Borrowing**	**% JSY beneficiaries**	**Mean OOPE in INR(SD)**	**Median OOPE in INR (IQR)**	**% Borrowing**	**% JSY beneficiaries**	
**Caste**	*p<0.01*	*–*	*p<0.04*	*p=0.35*	*p<0.01*	*–*	*p<0.01*	*p=0.11*	*p<0.01*	*–*	*p<0.02*	*p=0.69*	*–*
Scheduled caste	1493 (2045)	900 (500–1850)	41.3	44.7	3902 (4597)	3000 (1700–5000)	47.3	8.8	526 (1259)	300 (60–600)	33.4	3.8	16121
Scheduled tribe	1593 (2428)	1000 (500–2000)	24.6	45.1	3582 (3642)	2400 (1300–5000)	32.6	12.2	314 (669)	100 (0–400)	16.0	4.8	16591
Other backward caste	1465 (2189)	1000 (500–1800)	36.1	46.3	4369 (5193)	3090 (2000–5100)	37.0	7.2	493 (931)	250 (100–500)	28.7	2.5	**32049**
Others	2043 (2447)	1300 (600–2500)	26.1	34.7	4958 (5268)	3500 (2050–6000)	22.4	3.2	553 (980)	300 (44–600)	20.0	2.4	**16541**
**Education**	*p<0.01*	*–*	*p<0.01*	*p<0.06*	*p<0.01*	*–*	*p<0.01*	*p=0.84*	*p<0.01*	*–*	*p<0.01*	*p=0.88*	*–*
No schooling	1311 (1595)	850 (500–1550)	43.3	50.5	3204 (3644)	2200 (1300–3800)	53.1	6.4	408 (942)	200 (0–500)	29.3	2.5	**39087**
1-5 years	1494 (2280)	950 (500–1700)	37.7	44.8	3660 (4078)	2800 (1600–4417)	44.0	7.7	581 (997)	200 (0–500)	23.6	4.5	**12975**
6-11 years	1813 (2601)	1100 (530–2200)	26.7	38.8	4613 (5306)	3200 (2030–5300)	30.9	7.2	757 (1156)	300 (0–600)	17.0	4.6	**24455**
≥12 years	2241 (2736)	1500 (600–3000)	13.4	32.6	5727 (5817)	4250 (2500–7000)	14.1	4.5	408 (942)	500 (100–1000)	10.4	4.1	**652**3
**Wealth Index**	*p<0.01*	*–*	*p<0.01*	*p<0.01*	*p<0.01*	*–*	*p<0.01*	*p=0.45*	*p<0.01*	*–*	*p<0.02*	*p=0.99*	*–*
Poorest	1240 (1502)	800 (500–1500)	53.0	56.8	2877 (2837)	2050 (1100–3300)	64.2	8.0	353 (773)	200 (0–500)	31.6	3.3	18418
Second	1417 (1955)	900 (500–1600)	45.5	52.7	3086 (3428)	2200 (1200–3900)	54.9	8.7	425 (780)	200 (0–500)	27.9	3.5	18909
Middle	1534 (1905)	1000 (500–2000)	33.9	43.4	3701 (4570)	2600 (1544–4500)	47.7	9.4	496 (1275)	250 (0–500)	21.8	3.4	17841
Fourth	1792 (2680)	1100 (530–2150)	23.5	37.2	4211 (4954)	3040 (2000–5050)	36.0	8.2	596 (954)	300 (100–700)	17.9	3.1	16171
Richest	2162 (2926)	1200 (550–2800)	11.7	27.9	5523 (5692)	4070 (2500–6300)	16.0	3.2	789 (1278)	500 (200–1000)	12.7	2.5	11690
**Area**	*p=0.98*	*–*	*p<0.05*	*p<0.09*	*p<0.01*	*–*	*p<0.01*	*p=0.55*	*p<0.01*	*–*	*p=0.87*	*p=0.65*	*–*
Rural	1624 (2258)	1000 (520–2000)	35.8	45.7	4152 (4472)	3030 (1800–5100)	38.5	7.2	445 (860)	200 (0–500)	25.3	3.4	69715
Urban	1623 (2332)	1000 (468–2015)	22.5	34.2	5112 (6106)	3500 (2020–6000)	22.2	4.9	680 (1720)	494 (150–800)	24.1	2.3	13324
**Full ANC**	*p<0.01*	*–*	*p=0.22*	*p<0.07*	*p<0.01*	*–*	*p=0.09*	*p=0.58*	*p<0.01*	*–*	*p=0.31*	*p=0.12*	*–*
Yes	1862 (2473)	1100 (500–2300)	26.5	32.8	5340 (5220)	4020 (2300–6200)	26.1	5.7	707 (1277)	400 (0–1000)	19.6	8.4	12010
No	1561 (2213)	1000 (500–2000)	34.6	46.0	4054 (4949)	3000 (1700–5000)	36.5	7.9	449 (943)	200 (0–500)	25.6	2.9	71013
**Registered pregnancy and Got advice on institutional delivery**	*p<0.01*	*–*	*p=0.14*	*p=0.25*	*p<0.01*	*–*	*p<0.06*	*p=0.35*	*p<0.01*	*–*	*p=0.51*	*p=0.15*	*–*
Yes	1767 (2504)	1050 (500–2100)	29.2	40.1	4958 (5385)	3525 (2100–5748)	28.8	7.4	590 (1087)	300 (0–600)	21.9	6.2	34806
No	1413 (1861)	900 (500–1600)	38.5	47.8	3441 (4183)	2300 (1400–4100)	42.1	4.4	421 (922)	200 (0–500)	26.4	2.2	48235

INR: Indian National Rupees; SD: Standard Deviation; Borrowings: Households who borrowed money/sold property for meeting delivery expenses.

**Table 5:** **Mean & median out**-**of**-**pocket**-**expenditure (OOPE) of caesarean-section deliveries, percent borrowings and percent JSY-beneficiaries according to socio economic profile of women**

**Socio-demographic characteristics**	**C-section at public institution**				**C-section at private institution**				**Total women**
	**Mean OOPE in INR (SD)**	**Median OOPE in INR (IQR)**	**% Borrowing**	**% JSY beneficiaries**	**Mean OOPE in INR (SD)**	**Median OOPE in INR (IQR)**	**% Borrowing**	**% JSY beneficiaries**	
**Caste**	*p<0.01*	*–*	*p<0.01*	*p=0.11*	*p<0.01*	*–*	*p<0.01*	*p=0.29*	*–*
Scheduled caste	5335 (5635)	3294 (1500–8000)	53.1	35.2	12928 (8904)	11000 (7772–16064)	63.8	10.1	1205
Scheduled tribe	6060 (7519)	4000 (1627–8000)	32.5	30.9	15380 (14540)	12000 (7100–20000)	46.0	10.7	674
Other backward caste	5666 (6932)	3400 (1203–7497)	44.8	32.7	13764 (8320)	12600 (8700–18050)	53.5	9.4	3044
Others	6630 (7321)	5000 (2100–9300)	33.2	19.8	15220 (10408)	15000 (9003–20050)	31.6	3.9	2481
**Education**	*p<0.01*	*–*	*p<0.01*	*p=0.17*	*p<0.01*	*–*	*p<0.01*	*p=0.44*	*–*
No schooling	5506 (6396)	3200 (1300–7537)	56.2	28.0	11675 (8146)	10294 (5050–15500)	73.5	7.3	1338
1-5 years	4794 (5633)	3086 (1150–6012)	55.3	35.8	12206 (9358)	10300 (5400–15472)	68.9	10.6	785
6-11 years	5890 (6767)	4150 (1700–8038)	39.7	30.8	14013 (9172)	12700 (8816–18300)	48.9	8.9	3249
≥12 years	7197 (8043)	5023 (2150–10198)	21.1	22.2	16118 (10374)	15013 (10020–20100)	27.8	5.3	2180
**Wealth Index**	*p<0.01*	*–*	*p<0.01*	*p<0.01*	*p<0.01*	*–*	*p<0.01*	*p=0.13*	*–*
Poorest	5326 (7075)	2604 (1200–6756)	70.1	39.9	12363 (8205)	10600 (5042–17187)	82.8	14.8	401
Second	5304 (5803)	3452 (1500–7100)	63.7	39.3	12593 (8333)	10599 (7008–16057)	76.7	11.5	722
Middle	4926 (5038)	3267 (1300–7135)	54.2	38.4	12397 (8672)	10700 (6400–15500)	73.1	11.8	1211
Fourth	6247 (7306)	4726 (2000–8200)	40.7	28.0	13074 (8176)	12050 (7404–17000)	56.8	9.9	1997
Richest	6709 (7684)	5000 (2022–9247)	18.8	18.2	15723 (10546)	15000 (10000–20070)	28.6	4.3	3217
**Area**	*p=0.37*	*–*	*p<0.03*	*p=0.21*	*p<0.01*	*–*	*p<0.01*	*p=0.42*	*–*
Rural	5850 (6537)	4000 (1700–4000)	46.2	31.9	13549 (8808)	12300 (8000–18000)	55.8	8.8	4831
Urban	6117 (7497)	4400 (1510–8100)	30.8	23.5	15422 (10723)	15000 (10000–20020)	32.6	5.6	2721
**Full ANC**	*p=0.65*	*–*	*p=0.77*	*p=0.88*	*p<0.01*	*–*	*p=0.16*	*p=0.55*	*–*
Yes	5851 (6292)	4200 (1700–8000)	40.4	30.3	15233 (9563)	15000 (10000–20010)	41.2	9.4	2997
No	5982 (7178)	4000 (1510–8187)	41.8	28.5	13539 (9635)	12100 (7200–18050)	50.8	6.1	4548
**Registered pregnancy and Got advice on institutional delivery**	*p<0.07*	*–*	*p=0.15*	*p=0.88*	*p<0.01*	*–*	*p=0.10*	*p=0.15*	*–*
Yes	5805 (6280)	4050 (1700–8042)	39.1	29.1	14717 (9712)	13831 (9578–20000)	44.2	8.6	5822
No	6441 (8741)	4005 (1500–8400)	49.4	29.6	12605 (9173)	10623 (5500–16257)	55.5	3.7	1729

C-section: Caesarean-section; INR: Indian National Rupees; SD: Standard Deviation; Borrowings: Households who borrowed money/sold property for meeting delivery expenses.

## Discussion

In 2007–08, four years after the implementation of the JSY programme, half of all deliveries in India occurred at home. OOPE among women having institutional deliveries remained high, with considerable variation between the states/UTs. High OOPE due to institutional delivery forced one-third to half of the families to opt for borrowings, despite implementation of JSY programme to address this, reflecting both low use and the modest value for cash transfer within this programme. Even among women who had normal deliveries in public institutions, JSY use was less than 50% in 29 of the 34 states/UTs in India, highlighting scope for further improvement. Increased literacy and wealth were associated with a higher likelihood of an institutional delivery, but higher OOPE and no major variations in use of the JSY programme.

### How comparable are our results with other studies?

The Coverage Evaluation Survey (CES-2009) [12] report estimated mean expenditure for transporting a pregnant woman to facility in India at Rs. 192, while it was Rs. 322 in our study. A study done [25] in 12 districts of Uttar Pradesh reported average expenditure for institutional deliveries to be Rs. 1,179, which closely matches with our estimate for Uttar Pradesh (Rs. 1,246). Another cross-sectional survey from 12 districts of eight high focus – non NE states [26] (excluding Jammu & Kashmir and Himachal Pradesh) in 2010, reported average expenditure of an institutional delivery (excluding transportation) to be Rs. 1,028, while our mean expenditure of a normal institutional delivery in these eight states was Rs. 1,719 (SD=1,924). These variations in expenditures may be due to variations in the percentage of private hospital deliveries, 11% in our study and 5% in the reported study [26].

A comparison of our results (based on 2007/08 data) with those from the National Sample Survey Organization (NSSO) conducted in 2004 [27] suggests that OOPE to families for public and private institution delivery may have increased during this time period. In 2004, OOPE on a public, private and a home delivery respectively was Rs. 1,387, Rs. 6,094, and Rs. 428; while OOPEs in 2007/08 were Rs. 2,103, Rs. 7,245 and Rs. 466 respectively. There was no major increase in expenditure on home deliveries over this period. This data suggests that the JSY programme may not have offset increases in OOPE over that time period for many families.

Our findings suggest that the proportion of women opting for home deliveries in 2007/08 remains high (52%) in India; although a more recent (2009/10) estimate [12] found it to be 27%, suggesting that the JSY programme may have been successful in reducing the proportion of home deliveries since the DLHS-3 (2007–08) was conducted. Women from high focus-non NE states (where substantial portion of deliveries were at home) cited the following reasons for opting ‘home as the place of delivery’ in their previous pregnancy: not necessary to go to institution (33%); cost of institutional delivery was too much (25%); no time to go to institution (24%); better care at home (17%); institution too far/no transport (12%); lack of knowledge (7%); family did not allow (7%); not customary (7%); poor quality of service at institution (5%). This implies that barriers other than OOPE, including availability, accessibility, and lack of planning and cultural reasons need to be addressed to reduce home deliveries in India.

A cross-sectional survey [28] in 2008 found that the average amount paid by JSY beneficiaries to an institution for medicines and other services ranged from Rs. 299 in Madhya Pradesh to Rs. 1,638 in Orissa. These findings are consistent with ours, and imply that the JSY benefit is insufficient to cover expenditures incurred on delivery, thus, requiring many families to borrow money to pay for this. This is confirmed by our finding that rural families from high focus- non NE states had average additional expenditures of Rs. 544 and Rs. 4,761 for public and private institution deliveries respectively after receiving the JSY benefit (Rs 1400). Further, mean OOPE to families for normal deliveries in public institutions was more than the JSY-compensation amount of (Rs. 1,400) in five of the 10 high focus- non NE states.

### Study strengths and limitations

This study provides some of the first robust state-level estimates of OOPE for normal and c-section deliveries, the proportion of families required to borrow to meet these expenditures and the reach of JSY-programme, by location of delivery in India. One of the limitations of our study is OOPE to family on delivery care reported here are based on the figures recalled by women. Studies that gather expenditures of families from hospital records [4] are often more accurate as they are not influenced by recall or reporting bias. The current study only included direct expenses such as transportation and facility-based expenses. It did not include indirect expenses such as spending by women and families on food, other purchases during hospitalization/delivery, wages lost by women and family members during the delivery process and bribes/gifts. Results of this study must be seen in the light of limitations of the methods of DLHS-3 [29] which did not capture the reasons for variable implementation and use of JSY between different states [14], including eligibility guidelines, awareness of JSY programme, amount distributed, payment process, delays in payments to mothers and involvement of Associated Social Health Activists (ASHAs) in maternity care[25-27]. Before streamlining of JSY programme in 2007–08, there was very little change in the distribution of institutional deliveries during 2002–04 [30] and 2005–06 [11]. In 2009 proportion of institutional deliveries in India increased to 73% and JSY use increased to 33% [12], clearly implying that the coverage of the JSY has increased since 2007–08, and our findings are unlikely to reflect current JSY use and distribution of location of delivery, even though OOPE and family borrowings may not have changed markedly since 2007–08. Hence, ongoing evaluation of the JSY programme is essential to establish whether its reach and impacts on OOPE and family borrowings have improved.

### Policy implications

Our results highlight the ongoing high OOPE of Indian families for delivery/maternity care, resulting in 25-47% families in India having to borrow money to meet pregnancy/delivery related expenses. The OOPE burden was found to be especially high in: low wealth index, illiterate/less educated and low social group families and low per-capita income states [31]. The high levels of OOPE found, low reported use of the JSY programme and given that expenditures exceed the financial benefit of this programme for many families, suggest that the impact of programme on OOPE in 2007/08 appears to have been modest. Additional investment in the JSY programme, strengthening state-specific interventions targeting population groups most likely to avoid institutional care due to OOPE and providing support to families in financial planning for maternity care are likely to be required in order to meet the MDGs 4 and 5 in India.

## Conclusions

Our study highlights the ongoing high OOPE and impoverishing impact of institutional delivery care in India despite a high profile policy initiative seeking to address this issue. Additional investment in JSY and strengthening of state level implementation is required to increase coverage of JSY programme, reduce maternity related OOPE, reduce delivery associated borrowings and increase the proportion of institutional deliveries in India. Such an investment is vital to accelerate progress towards achievement of MDGs 4 and 5.

### Endnotes

^a^12 cases out of 36,536 were excluded from analysis as outliers

^b^14 cases out of 83,524 were excluded from analysis as outliers

## Abbreviations

ANC: Antenatal care; ANOVA: Analysis of variance; ASHA: Accredited Social, Health Activist; AYUSH: Ayurveda, Yoga, Unani, Siddha & Homeopathy; BPL: Below Poverty Line; CES: Coverage Evaluation Survey; C-section: Caesarean section; DLHS: District Level Household and facility Survey; GBP: British Pound sterling; INR: Indian Rupee; IQR: Inter-quartile range; JSY: Janani Suraksha Yojana; MDG: Millennium Development Goals; MMR: Maternal Mortality Ratio; NE: North Eastern; NGO: Non-governmental organisation; NRHM: National Rural Health Mission; NSSO: National Sample Survey Organisation; OOPE: Out of pocket expenditure; SD: Standard deviation; SPSS: Statistical Package for Social Sciences; U5MR: Under five mortality rate; UK: United Kingdom; UT: Union-Territory.

## Competing interests

The authors have no financial benefits or competing interests related to this work.

## Author’s contributions

HRM led conceptualization, literature review, conducted all analysis, and led manuscript development and finalization. MK assisted with literature review. AK assisted with conceptualization and analysis. CM assisted with analytic approach, literature review, and editing of the manuscript. All the authors participated as described above and all read and approved this final submitted manuscript.

## Pre-publication history

The pre-publication history for this paper can be accessed here:

http://www.biomedcentral.com/1471-2458/12/1048/prepub
